# Editorial Department

**Published:** 1857-12

**Authors:** H. P. Strong

**Affiliations:** Beloit, Wis.


"Is the effusion of blood from the ear a pathognomonic sign of fracture of the base of the cranium?"

In the report of the proceedings of the Buffalo Medical Association, reported in the October number of your journal, I find the above question asked by Dr. Lemon. As the minutes of three cases of effusion are now at hand which have fallen under my observation, I will give them that they may contribute something to settle the question.

Case I. Dr. S., æt. 50, was thrown from his carriage in August, 1857; I saw him one half hour after the injury. Says he was thrown some distance and struck upon the right side of his head, face and shoulder. I found the integument over the right os frontis much bruised, extending to the eye and side of the face. The shoulder was badly sprained. There was *hæmorrhage from the ear* for some three hours after the injury. Reäction was established in about three hours from the time of the injury. His recovery was rapid, without coma, or a symptom of effusion or compression.

Case II. P. McC., æt. about 40, while engaged in an Irish fight, in Aug., 1857, was struck a blow, over the right os frontis, with a large piece of wood; as he reached the ground, the back side of his head came in collision with a bar of iron on the railroad track. When first seen he was partially comatose. He remained in this condition about one hour. There were no symptoms of osseous fracture, yet there was quite profuse *hæmor-*

*rhage from the ear* for one hour. He was at his work again in two days, feeling as well as ever, save a "sore head."

CASE III. P. M., æt. 30, was knocked down in a street fight, in Sept., 1857, and severely beaten about the head by his assailants, with their fists and boots, until he was unconscious. When first seen he presented all the appearances of having received a severe shock. Smart reäction took place in one hour.

There was *hæmorrhage from both ears*, though not to any great extent, nor of so long continuance as in the preceding case. On the third day after the date of injury he was about town at his usual work.

In all these cases I made a careful examination to find injuries of the ear, but in neither case could I detect any. The hæmorrhage seemed to proceed from internal vessels.

Yours, &c.,

BELOIT, Wis., Nov. 9th, 1857. H. P. STRONG.

